# Breathing maneuvers may elicit a stronger myocardial vascular response than clinical adenosine protocols

**DOI:** 10.1186/1532-429X-16-S1-P49

**Published:** 2014-01-16

**Authors:** Kady Fischer, Dominik P Guensch, Matthias G Friedrich

**Affiliations:** 1 Philippa & Marvin Carsley CMR-Centre at the Montreal Heart Institute, Montreal, Quebec, Canada; 2 Department Anesthesiology and Pain Medicine, Inselspital Bern, University of Bern, Bern, Switzerland

## Background

Adenosine is one of the currently used agents for pharmacological vasodilation protocols used in imaging myocardial perfusion deficits. Yet, its clinical utility is limited by cost, need for i.v. access, and by side effects such as dyspnea and AV block, requiring the presence of a trained physician during administration. Recently, breath holds have been proposed as a potential alternative to adenosine administration; yet, the vasodilatory response has not been compared with adenosine as a standard vasodilatory agent. We investigated the use of breath-holds to induce vasodilation in healthy volunteers in direct comparison to the reference adenosine using oxygenation-sensitive (OS)-CMR, which allows for non-invasive monitoring of changes in myocardial tissue oxygenation. We combined a period of hyperventilation with a long voluntary breath-hold to maximize the range of the inducible vasodilatory response.

## Methods

We studied 19 healthy volunteers (mean age 43 ± 4 y) using a clinical 3T MRI system. OS-CMR images were acquired in one mid-ventricular short axis slice using an ECG-triggered balanced SSFP sequence. Volunteers hyperventilated for 60 s aiming for a rate of 40 breaths/min followed by a maximal long breath-hold at end-expiration (HVBH). OS-CMR images were acquired continuously throughout the long breath-hold at an acquisition rate of 1 measurement every 4 heart beats until the participant voluntarily commenced breathing. Single measurement acquisitions were obtained for a baseline image and during (3.5 minutes after start) adenosine infusion (140 μg/kg/min, i.v.). Systolic images were analyzed for the global myocardial signal intensity (SI) change in comparison to baseline, expressed as % change. The breath hold was assessed at two time points; the end of the breath-hold and the when the maximal SI occurred. On a questionnaire, volunteers ranked the maneuvers based on difficulty to perform.

## Results

The mean duration of the HVBH was 74 s (± 7 s) with a final change in SI of 12.8%* (± 2.0), but a peak SI of 18.1%* (± 3.9) was reached after 41 s (± 4). In comparison, the change after adenosine was significant but yielded a change of 2.8%(± 1.2)* only. Both the final and peak SI values were significantly greater than adenosine (*p < 0.05, n = 19). There was not a significant difference in difficulty between the two maneuvers from the questionnaire but 5 volunteers experienced adverse effects with the HVBH that all disappeared with normal breathing, whereas 11 volunteers (58%) experienced adverse effects from adenosine, with 3 saying the effects persisted even after the drug was stopped.

## Conclusions

A breathing maneuver combining hyperventilation with a long breath-hold may elicit a stronger vasodilatory response than a standard clinical infusion of adenosine and may be a simpler, cheaper, and more effective approach to assess the vascular response in patients with suspected coronary artery disease.

## Funding

Funding is provided by the Montreal Heart Institute Foundation and the Canadian Foundation for Innovation.

**Figure 1:**
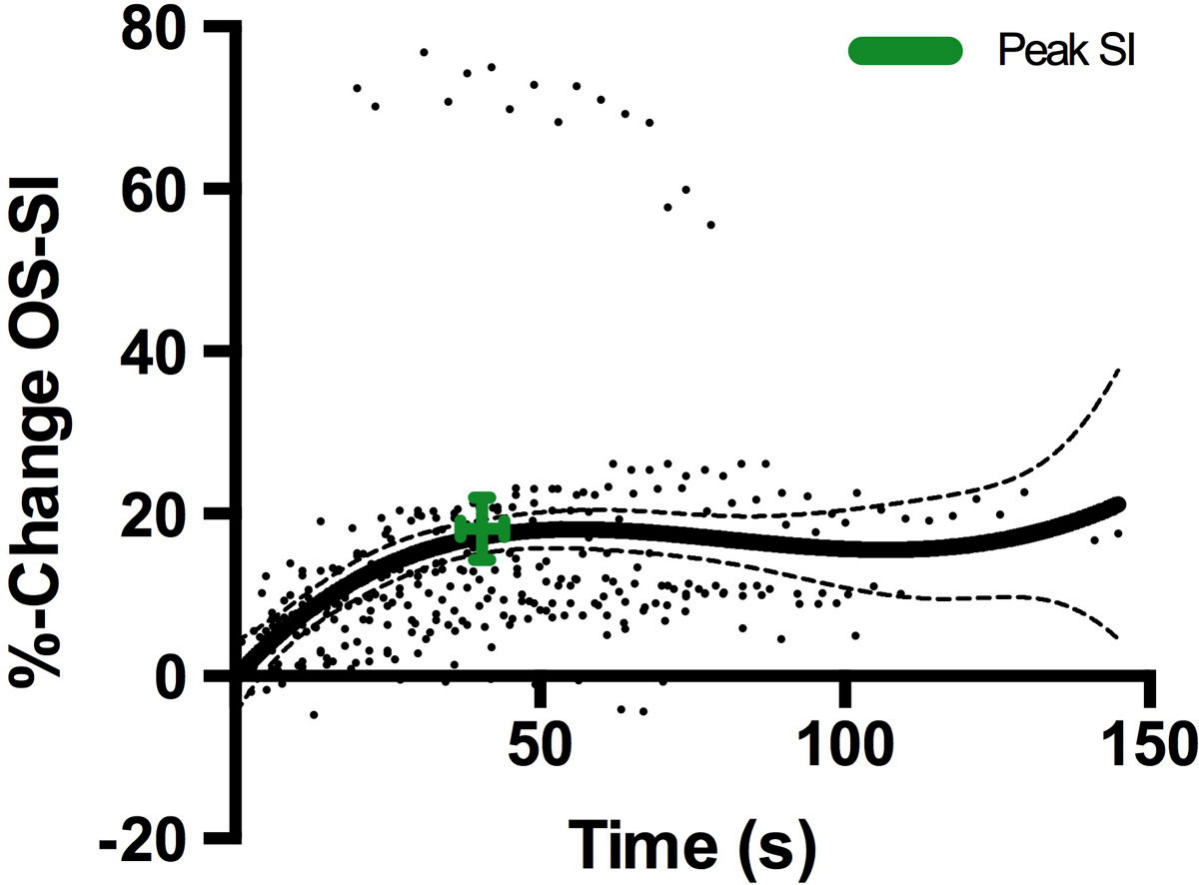
**The %-change SI over the time duration of the long breath-hold plotted with non-linear regression and 95% confidence intervals (n = 19)**. The range displayed in green demonstrates the mean peak SI and mean time this was achieved.

**Figure 2:**
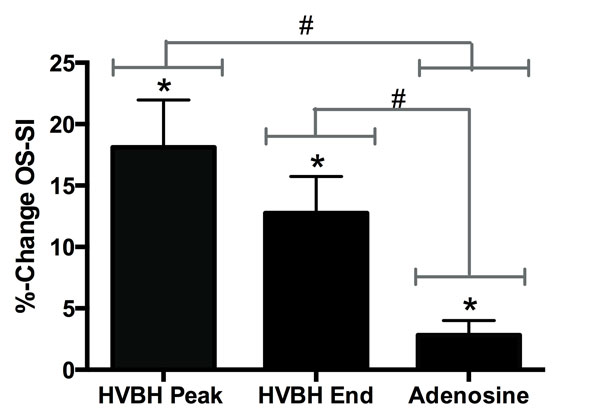
Mean ± SEM %-change OS-SI from the peak of the HVBH, the end of the HVBH and adenosine (*p < 0.05, n = 19). Adenosine was significantly lower then both breath-hold data points (# < 0.05).

